# Mendelian randomization study on the causal effects of tumor necrosis factor inhibition on coronary artery disease and ischemic stroke among the general population

**DOI:** 10.1016/j.ebiom.2022.103824

**Published:** 2022-01-22

**Authors:** Xiaoying Kang, Tong Jiao, Haiyang Wang, John Pernow, Karin Wirdefeldt

**Affiliations:** a Department of Medical Epidemiology and Biostatistics, Karolinska Institutet, Stockholm, Sweden; b Unit of Cardiology, Department of Medicine, Karolinska Institutet, Stockholm, Sweden; c Department of Vascular Surgery, The First Affiliated Hospital of Guangzhou Medical University, Guangzhou, China; d Unit of Cardiology, Department of Medicine, Karolinska Institutet, Stockholm, Sweden; e Department of Cardiology, Karolinska University Hospital, Stockholm, Sweden; f Department of Clinical Neuroscience, Karolinska Institutet, Stockholm, Sweden

**Keywords:** Tumor necrosis factor inhibition, Coronary artery disease, Ischemic stroke, Mendelian randomization

## Abstract

**Background:**

Tumor necrosis factor (TNF) is a potent inflammatory cytokine that has been causally associated with coronary artery disease (CAD) and ischemic stroke (IS), implying opportunities for disease prevention by anti-TNF therapeutics.

**Methods:**

Leveraging summary statistics of several genome-wide association studies (GWAS), we assessed the repurposing potential of TNF inhibitors for CAD and IS using drug-target Mendelian randomization (MR) design. Pharmacologic blockade of the pro-inflammatory TNF signalling mediated by TNF receptor 1 (TNFR1) was instrumented by four validated variants. Causal effects of TNF/TNFR1 blockade on CAD (N_case/control_ upto 122,733/424,528) and IS (N_case/control_ upto 60,341/454,450) were then estimated via various MR estimators using circulating C-reactive protein (CRP; N_GWAS_=204,402) as downstream biomarker to reflect treatment effect. Associations of a functional variant, rs1800693, with CRP, CAD and IS were also examined.

**Findings:**

No protective effect of TNF/TNFR1 inhibition on CAD or IS was observed. For every 10% decrease of circulating CRP achieved by TNF/TNFR1 blockade, odds ratio was 0.98 (95% confidence interval [CI]: 0.60-1.60) for CAD and 0.77 (95% CI: 0.36-1.63) for IS. Findings remained null in all supplement analyses.

**Interpretation:**

Our findings do not support TNFR1 as a promising target for CAD or IS prevention among the general population. Future research is warranted to investigate whether the detrimental effect of circulating TNF on CAD and IS might be counteracted by modulating other relevant drug targets.

**Funding:**

No.

**Research in context**

**Evidence before this study**

PubMed and Google Scholar were searched to extract literature containing “tumor necrosis factor” and “cardiovascular disease” in title. Based on the results, tumor necrosis factor has been associated with many cardiovascular events by genetic, epidemiological and experimental data. Numerous clinical and animal studies also suggested the opportunity to prevent cardiovascular diseases or their underlying risk factors with anti-tumor necrosis factor strategies. The causal effect of tumor necrosis factor on coronary artery disease and ischemic stroke, was recently reported by a Mendelian randomization study as well.

**Added value of this study**

The present work followed up the causal effect revealed by the recent Mendelian randomization study and, for the first time, provided supplement data about the potential benefit of inhibiting the proinflammatory signalling pathway associated with this pathogenic cytokine on coronary artery disease and ischemic stroke. These results are informative to the future development of novel clinical therapeutics and will enhance our understanding the pathogenic role of tumor necrosis factor and its relevant signalling pathways in cardiovascular events.

**Implications of all the available evidence**

The detrimental effect induced by tumor necrosis factor on coronary artery disease and ischemic stroke is multifactorial and cannot be simply reversed by deactivating the tumor necrosis factor receptor 1-mediated proinflammatory signalling among the general population.

Alt-text: Unlabelled box

## Introduction

Tumor necrosis factor (TNF) is a potent inflammatory cytokine involved in a broad spectrum of biological processes. TNF can exist in two forms: synthesized as a transmembrane protein, TNF can be cleaved by the TNF-α converting enzyme (TACE) into a soluble variant.^1^ Receptor binding is a crucial step for TNF signalling transduction. TNF receptor 1 (TNFR1), a death receptor ubiquitously expressed by most cell types, binds to both forms of TNF and triggers different downstream effectors that ultimately lead to both cytoprotective and apoptotic effects.^2^^,^^3^ In contrast, TNFR2 locates in restricted cell types (e.g. endothelial cells and immune cells) and is activated preferentially by transmembrane TNF to induce a mostly pro-survival signalling.^3^^,^^4^ Instead of acting independently, TNFR1 and TNFR2 crosstalk with each other, maintaining the delicate balance between the two signalling pathways under the regulation of TNF receptor associated factors (TRAFs).^5^

Historically known for an integral role in host defense, TNF is recognized more recently for its deleterious effect in several inflammatory and autoimmune diseases, which prompted the development of anti-TNF therapeutics. To date, five TNF inhibitory drugs – infliximab, etanercept, adalimumab, golimumab and certolizumab – have been approved to treat rheumatoid arthritis and Crohn disease etc. in clinical practice.^6^ The observed clinical benefits also encouraged an abundance of contemporary research to probe into the repurposing potential of anti-TNF strategy in other relevant conditions, such as Dupuytren's disease.^7^^,^^8^

Cardiovascular diseases (CVDs) have been linked with TNF by genetic and epidemiological evidence.^9^, ^10^, ^11^, ^12^, ^13^, ^14^, ^15^, ^16^ Multiple hypotheses of the biological mechanisms through which TNF may contribute to CVDs were also proposed by the accumulating experimental studies on animal models.^17^, ^18^, ^19^ Recently, using Mendelian randomization (MR) design, Yuan et al. reported a causal association of circulating TNF with the risk of coronary artery disease (CAD) and ischemic stroke (IS) among the general population, indicating TNF signalling as a promising target for the primary prevention of these diseases.^20^ Therefore, we followed up with this interesting finding and examined whether TNF inhibition may lower the risk of CAD and/or IS.

## Methods

### Study design

The causal effects of TNF inhibition on CAD and IS were examined in a “drug-target MR” design using GWAS summary statistics. Drug-target MR is developed on the basis of the classical MR design, which uses genetic variation as instrumental variable to test for causality between a modifiable exposure and an outcome.^21^ Because alleles segregate independently during the gamete formation, the genetic instrumentation of exposure in MR studies is considered to be analogous to the randomization of intervention in a randomized controlled trial and is rarely influenced by any confounding factors that appear after birth.^22^ For these reasons, MR findings are usually robust to confounding or reverse causation, both of which are common concerns in observational epidemiological studies.

**Figure 1:**
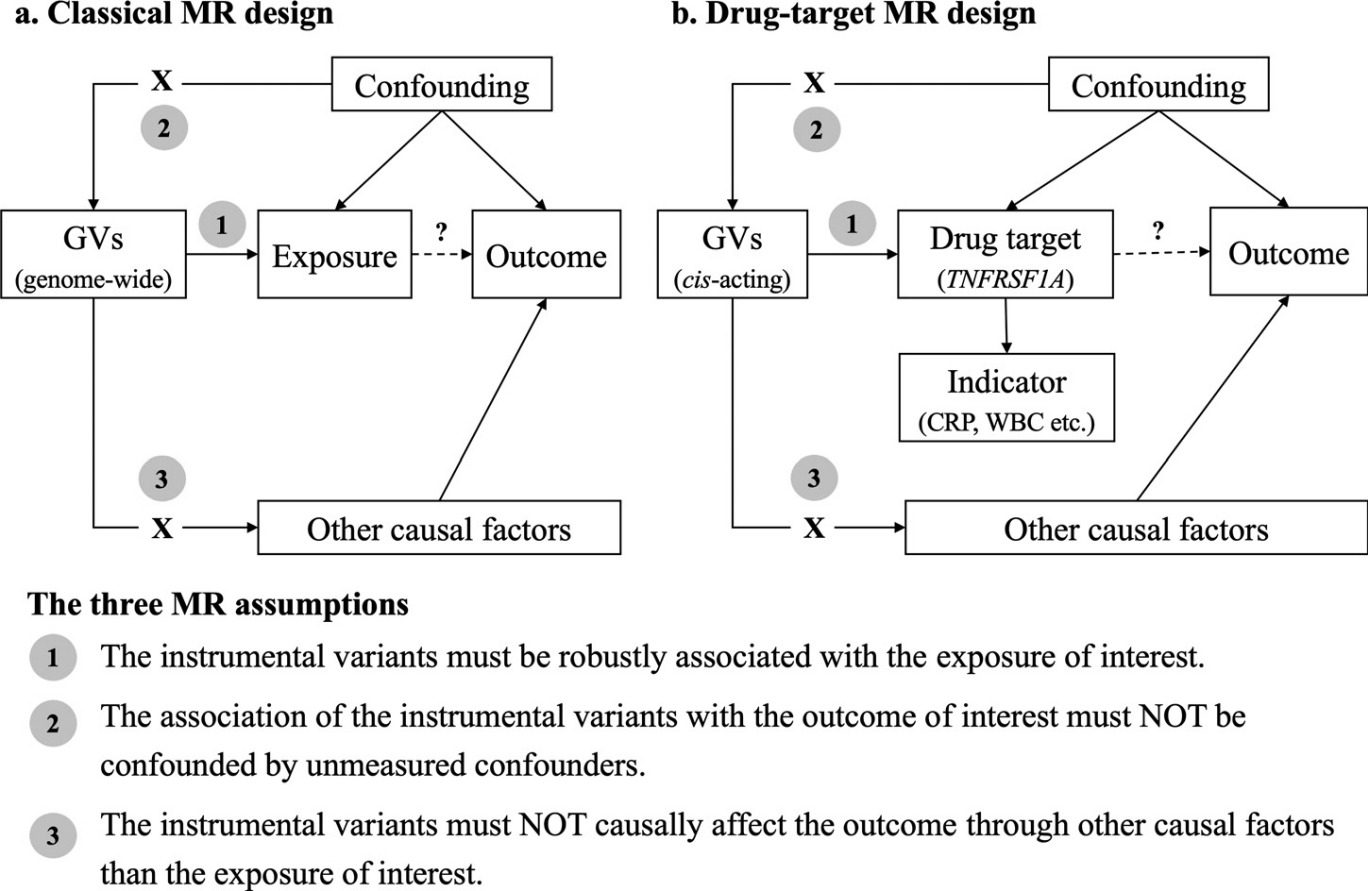
Directed acyclic graphs for the classical (a) and the “drug-target” (b) Mendelian randomization designs. The arrows denote causal relations between two variables, pointing from the cause to the effect. The causal pathway is blocked if “X” is placed in the arrowed line. The main research question about the causal pathway between the exposure/drug target and the outcome is distinguished from other pathways by a dotted line and question mark above it. (**a**) The classical MR design aims to investigate whether a biomarker or risk factor (exposure) plays a causal role in the disease outcome of interest and typically selects the genetic instruments from genome-wide per statistical evidence on association (e.g. by requiring association *p*-value < 5 × 10^−8^). (**b**) The drug-target MR design, developed from the classical MR, seeks to estimate the causal effect of modifying a specific drug target on a disease outcome. For this purpose, the drug-target MR design considers only genetic variants located in the vicinity of the specific gene encoding the drug target of interest as instrumental variables. *Abbreviations*: MR, Mendelian randomization; GV, genetic variants; *TNFRSF1A*, tumor necrosis factor receptor superfamily 1A; CRP, C-reactive protein; WBC, white blood cell counts.

As illustrated in Figure 1, three assumptions are crucial to the validity of MR studies. First, the genetic instruments must be robustly associated with the exposure of interest. Second, the associations of the genetic instruments with the outcome of interest must not be confounded by unmeasured confounding. Third, the genetic instruments must not causally affect the outcome through other factors than the exposure of interest. In classical MR where the research aim is mainly to test whether the exposure (such as a biomarker or environmental risk factor) plays a causal role in a disease outcome, genetic instruments are selected from the genome-wide based on statistical evidence on the association with the exposure (e.g. by requiring association *p*-value < 5 × 10^−8^) (Figure 1a). In contrast, drug-target MR seeks to estimate the causal effect of modifying a drug target on a disease outcome and therefore considers only genetic variants located in the vicinity of the specific gene encoding the drug target of interest as instrumental variables (Figure 1b).^23^ Since regional variants mostly regulate their own gene expression but rarely influence other gene products, such restriction by genomic location not only strengthens the reliability of the genotype-phenotype association (enhancing the first MR assumption) but also minimizes the possibility of violating the other two MR assumptions.^24^

Hence, to investigate TNF signalling as a potential target for CAD and IS, we implemented the drug-target MR design in the present work (Figure 1b). Specifically, we defined the exposure of interest as the pharmacologic blockade of TNF signalling mediated specifically by TNFR1. The selective choice of TNFR1 as the drug target was explained in our earlier publication.^25^ Briefly, the pathogenic role of TNF in CAD and IS is mainly thought of as a consequence of its pro-inflammatory effect activated by the binding of TNF to TNFR1.^19^^,^^26^ Moreover, the other receptor, TNF receptor 2, has been increasingly demonstrated to be cardio- and neuro-protective and should therefore not be antagonized in novel anti-TNF strategies.^4^^,^^27^, ^28^, ^29^ The two outcomes, CAD and IS, were examined separately.

### Instrumentation of TNF/TNFR1 Inhibition

A total of 4 single nucleotide polymorphisms (SNPs) that have been previously validated by Kang et al. were used to instrument the pharmacologic inhibition of TNF/TNFR1 pathway.^25^ In essence, the instrumental variants were selected based on two criteria. First, the SNPs must be located within or ±1kb around *TNFRSF1A* (chromosome and base pairs per GRCh37, 12:6437923-6451280)*,* the gene encoding TNFR1. Second, the SNPs must be associated with circulating C-reactive protein (CRP), a sensitive inflammatory biomarker that is commonly used as endpoint in the clinical assessment of anti-TNF efficacy.^30^ Of note, CRP was only introduced as a downstream marker to quantitatively indicate the magnitude of the anticipated anti-inflammatory treatment effect following TNFR1 antagonism, and it does not necessarily need to mediate the putative causal pathway between the TNF/TNFR1 signalling and the disease outcomes (Figure 1). To minimize the possibility of chance finding for CRP associations, we further required genetic association with two additional inflammatory biomarkers, white blood cell count (WBC) and mean platelet volume (MPV), for inclusion. Throughout the analyses, we re-scaled the CRP association statistics (coefficient and its standard error) of included instrumental variants to reflect the causal effects from every 10% decrease in circulating CRP level achieved by blocking TNFR1.^31^

### Trait measurement and GWAS data

Genetic associations with the serum concentration of CRP, measured by immune assay as mg/L, came from a meta-analysis of 204,402 European individuals enrolled in the Cohorts for Heart and Aging Research in Genomic Epidemiology (CHARGE) Inflammation Working Group.^32^ GWAS of WBC and MPV, measured by complete blood count technique, was based on 173,480 individuals of European ancestry included in the UK Biobank, the UK BiLEVE study and the INTERVAL study.^33^ Associations of the instrumental SNPs with IS were extracted from the multi-ancestry GWAS of 60,341 cases and upto 454,450 controls in the MEGASTROKE consortium, where the World Health Organization definition for stroke was used in conjunction with clinical and imaging criteria to define IS.^34^ In line with the statement from the Stroke Council of the American Heart Association/American Stroke Association, such comprehensive definition integrated the clinical symptoms, pathological/imaging evidence and other clinical data on risk factors and/or family history and may achieve a diagnostic accuracy of 95%.^35^^,^^36^ For CAD, we used the SNP associations meta-analyzed from two sources of European participants: the discovery sample nested in the UK Biobank cohort (34,541 cases and 261,984 controls) and the replication sample from CARDIoGRAMplusC4D consortium (88,192 cases and 162,544 controls).^37^ Patients with CAD in the UK Biobank was mainly identified from the linked national registered using the International Classification of Diseases 10 codes I21-I25 and surgical codes (Office of Population Censuses and Surveys Classification of Interventions and Procedures version 4) K40-K46, K49, K50 and K75.^37^ For the CARDIoGRAMplusC4D consortium, an inclusive criteria was adopted to determine CAD status, as specified by the Coronary Artery Disease Consortium.^38^^,^^39^

### Statistical analysis

Following the analytical protocol we previously validated, the statistical analysis was performed in three sections.^25^ Using the independent single SNP (*r*^2^ < 0.001) with smallest association *p* values for CRP (rs767455, *F* statistics = 28.9; Table 1), we fit the main model with Wald ratio estimator. In the secondary MR analysis, 3 partially correlated SNPs (*r*^2^ < 0.8), including rs767455, were included in the inverse variance weighting-based estimation to improve statistical power. Numerical instabilities due to the inclusion of correlated variants was controlled for by integrating principal component analysis.^40^ Finally, genetic associations of the CRP-lowering allele (T/C) of rs1800693, a variant with functional similarity to TNF antagonist, with the outcome variables were also examined.^41^ Validity of all the four analyzed SNPs and the analytical protocol was previously shown by positive control analysis on Crohn disease, ulcerative colitis and multiple sclerosis, and can be found in our previous publication.^25^

**Table 1:** The included instrumental variants and their genetic associations with inflammatory markers and outcome traits.

	rs767455	rs4149570	rs4149577	rs1800693
CRP-decreasing allele	C	C	A	C
Other allele	T	A	G	T
*Associations with inflammatory biomarkers: β (standard error), p-value*				
CRP	-0.023 (0.004), 7.8e-8	-0.016 (0.004), 2.4e-4	-0.018 (0.004), 2.5e-5	-0.024 (0.004), 9.2e-8
WBC	-0.011 (0.004), 4.2e-3	-0.015 (0.004), 1.3e-4	-0.015 (0.004), 3.2e-5	-0.010 (0.004), 9.1e-3
MPV	-0.017 (0.004), 3.7e-6	-0.013 (0.004), 3.6e-4	-0.011 (0.004), 2.4e-3	-0.016 (0.004), 1.3e-5
*Associations with outcome traits: β (standard error), p-value*				
CAD	0.001 (0.006), 0.93	-0.006 (0.005), 0.29	-0.006 (0.005), 0.27	-0.0003 (0.006), 0.96
IS	0.006 (0.009), 0.49	0.005 (0.009), 0.56	0.004 (0.009), 0.66	0.006 (0.009), 0.50

*Abbreviations*: CRP, C-reactive protein; WBC, white blood cell count; MPV, mean platelet volume; TNF, tumor necrosis factor; CAD, coronary artery disease; IS, ischemic stroke.

Causal estimates of TNF/TNFR1 inhibition on the binary disease outcomes from the primary and secondary MR models were presented as odds ratios (ORs) and 95% confidence intervals (CIs) for every 10% decrease of circulating CRP. Statistical significance was determined as two-sided *p* < 0.05 without multiple correction. The statistical analyses were carried out in R version 4.0.5 (2021-03-31) using *TwoSampleMR* package.^42^

### Ethics

No ethical approval was required for the present study, since all analyses were only based on publicly available summary statistics without accessing individual-level data. The included GWAS studies all received informed consent from the study participants and have been approved by pertinent local ethical review boards.

### Role of funders

The study received no financial support.

## Results

No evidence was observed for a causal effect of TNF/TNFR1 inhibition on the risk of CAD or IS (Table 2). In the main model using the independent single SNP (rs767455) as genetic instrument, every 10% decrease of circulating CRP was associated with an OR of 0.98 (95% CI: 0.60, 1.60) for CAD and 0.77 (95% CI: 0.36, 1.63) for IS. The findings remained null in the secondary model including two additional SNPs: the inverse variance weighting-based OR and 95% CI for CAD and IS were 1.01 (0.62, 1.65) and 0.77 (0.36, 1.65), respectively. Similarly, we found no genetic associations of the functional variant rs1800693 with either CAD (association coefficient: -0.0003; standard error: 0.006; *p* value: 0.96) or IS (association coefficient: 0.006; standard error: 0.009; *p* value: 0.50).

**Table 2:** MR estimates for the causal effects of TNF/TNFR1 inhibition on coronary artery disease and ischemic stroke.

Outcome	Wald ratio		Inverse variance weighting	
	OR (95% CI)	*P*	OR (95% CI)	P
Coronary artery disease	0.98 (0.60, 1.60)	0.93	1.01 (0.62, 1.65)	0.97
Ischemic stroke	0.77 (0.36, 1.63)	0.49	0.77 (0.36, 1.65)	0.50

*Abbreviations*: MR, Mendelian randomization; TNF, tumor necrosis factor; TNFR1, tumor necrosis factor receptor 1; OR, odds ratio; CI, confidence interval.

All results are expressed as odds ratios per a long-term 10% reduction in circulating level of C-reactive protein to reflect the anticipated anti-inflammatory effect due to TNF/TNFR1 blockade.

## Discussion

In the present work, we tested the causal effects of anti-TNF on CAD and IS susceptibility using MR design and found no evidence for any benefits of inhibiting the TNF/TNFR1 signalling pathway on either disease. Our study is based on the most updated GWAS summary data and robust study design, and our results remain consistently null throughout the analyses.

### Results interpretation

In light of the prior evidence on the causal associations of circulating TNF with increased risks of CAD and IS, the absence of benefits of TNF/TNFR1 blockade we observed tends to be counterintuitive at first glance.^20^ Nonetheless, to conclude from the two seemingly contradictory findings, a couple of points should be discussed.

First, it is worth noting that the causal estimate from MR study is an averaged effect across all population and their entire lifespans, which means that if TNF/TNFR1 blockers play opposing effects among subpopulations with different characteristics, the ultimate MR estimate can be neutralized towards the null.^43^ This is of particular clinical relevance because paradoxical results have indeed emerged from studies of different patient groups.^18^ For instance, anti-TNF therapy is often linked with lower risk of cardiovascular events among patients with autoimmune conditions like rheumatoid disease^7^^,^^19^^,^^44^, but shows no or even adverse effects for heart failure or myocardial infarction at high doses.^7^^,^^45^, ^46^, ^47^ Therefore, rather than precluding any potential cardioprotective effects of TNF inhibitory drugs, our findings support the contemporary perception that the success of anti-TNF strategy depends heavily on who and when to treat and at what doses.^6^

Second, since the TNF-associated CVD risks cannot be inversed by blocking TNFR1, it is reasonable to speculate that the pathogenic contribution of TNF is underpinned by mechanisms beyond the TNF/TNFR1 pathway and its downstream effects. One possible process is the TNF/TNFR2 signalling, for which a cardioprotective and immunoregulatory property has been demonstrated by mounting preclinical data.^28^^,^^48^, ^49^, ^50^, ^51^ Indeed, monoclonal antibodies that specifically activate TNFR2 have already been advocated as a novel therapeutic strategy for conditions including CVDs.^19^^,^^52^ Given the fact that TNFR2 has higher affinity to transmembrane TNF, supressing TACE, the enzyme that truncates membrane-bound TNF into soluble cytokine, may act similarly as the TNFR2 agonists.^1^, ^2^, ^3^^,^^53^ However, due to the lack of valid downstream effectors, we were unable to predict the effect of boosting TNF/TNFR2 signalling via either directly upregulating the receptor or indirectly modulating TACE on CAD or IS. Future research is therefore warranted to probe into these drug targets using alternative approaches.

Another point to emphasize is that both CAD and IS are complex diseases that are attributable to polygenic determinants and gene-environment interaction.^54^, ^55^, ^56^, ^57^ This means that an effective preventative intervention for CAD or IS might not be achieved monogenically but require a multifactorial approach with possible individualized considerations. It is therefore conceivable that blocking TNF signalling might work in conjunction with other interventions, such as lifestyle adjustment and mitigation of comorbidities, despite the evident role of TNF in causing the two diseases.^20^^,^^58^^,^^59^

### Caveats and limitations

Our study has several limitations. First, as discussed above, we were unable to identify the potential beneficiary population for TNF/TNFR1 inhibition due to data unavailability. Restricted by the knowledge gaps, we were also not able to evaluate the putative impact of targeting TNFR2 on CAD or IS. Besides, since a total of three correlated SNPs were included as instrumental variants, we performed the MR analyses with only two methods and did not manage to test for violation of MR assumptions using alternate estimators, such as MR-Egger. Such methodological caveat may to some degree jeopardize the internal validity of our study; nevertheless, the instrument validity of these selected SNPs has been previously validated by Kang et al. and findings in the present work were highly consistent across different models.

## Conclusions

In summary, the results from Yuan et al.’s and our work collectively imply that the pathogenic role of TNF in CAD or IS is complex and cannot be simply reversed by deactivating the TNFR1-mediated pathway among the general population.

## Declaration of interests

Nothing to disclose.
